# Supplementary material for: Copper supplementation mitigates Parkinson-like wild-type SOD1 pathology and nigrostriatal degeneration in a novel mouse model
Source: Acta Neuropathol Commun. 2025 Jun 25;13:133. doi: 10.1186/s40478-025-02048-2 (PMC12188662; doi:10.1186/s40478-025-02048-2)
Supplement: Supplementary file 2 — Supplementary Material 2 [file 40478_2025_2048_MOESM2_ESM.pdf]

SUPPLEMENTARY INFORMATION – UNCROPPED BLOTS

Copper supplementation mitigates Parkinson-like wild-type SOD1 pathology and nigrostriatal degeneration in a novel mouse model

Authors: Benjamin D. Rowlands[1#], Benjamin G. Trist[1#], Conor Karozis[1], Greta Schaffer[1], David Mor[1], Richard Harwood[2], Sarah A. Rosolen[1], Veronica Cottam[1], Freyja Persson-Carboni[1], Miriam Richardson[1], Anne A. Li[1], Michael P. Gotsbacher[3], Amr H. Abdeen[1], Rachel Codd[3], Kay L. Double[1*]

Affiliations:

1. *Brain and Mind Centre and School of Medical Sciences (Neuroscience), Faculty of Medicine and Health, The University of Sydney, Sydney, New South Wales 2006, Australia*
2. *Sydney Microscopy and Microanalysis, The University of Sydney, Sydney, New South Wales 2006, Australia*
3. *School of Medical Sciences (Molecular Biomedicine), Faculty of Medicine and Health, The University of Sydney, Sydney, New South Wales 2006, Australia*

* Correspondence should be addressed to Professor Kay L. Double, Brain and Mind Centre, 94-100 Mallett Street, Camperdown, NSW 2050, Australia. E-mail: kay.double@sydney.edu.au.

[#] These authors contributed equally.

Table 1. Gel loading order for midbrain tissue extracts subjected to immunoblotting for SOD1 and GAPDH. Lane 1 of each gel was loaded with molecular weight ladder (Precision Plus Protein Dual Xtra Standards), while lane 2 was loaded with a loading control (LC) comprised of an equal mixture of every sample.

Gel	Lane	Sample	Gel	Lane	Sample
1	2	LC	5	3	LC
1	3	80	5	4	94
1	4	93	5	5	108
1	5	104	5	6	110
1	6	112	5	7	115
1	7	121	5	8	116
1	8	124	5	9	154
1	9	125	5	10	155
1	10	136	5	11	163
1	11	145	6	2	LC
1	12	166	6	3	78
1	13	188	6	4	95
2	2	LC	6	5	114
2	3	82	6	6	117
2	4	89	6	7	118
2	5	91	6	8	120
2	6	92	6	9	141
2	7	101	6	10	142
2	8	140	6	11	160
2	9	147	6	12	161
2	10	156	7	2	LC
2	11	177	7	3	75
2	12	176	7	4	76
3	2	LC	7	5	79
3	3	82	7	6	85
3	4	89	7	7	98
3	5	91	7	8	107
3	6	92	7	9	109
3	7	101	7	10	143
3	8	140	7	11	162
3	9	147	7	12	169
3	10	156	7	13	187
3	11	177	8	2	LC
4	2	LC	8	3	77
4	3	86	8	4	96
4	4	87	8	5	97
4	5	90	8	6	99
4	6	102	8	7	100
4	7	105	8	8	113
4	8	106	8	9	119

4	9	135	8	10	144
4	10	137	8	11	167
4	11	139	8	12	168
4	12	157			

a

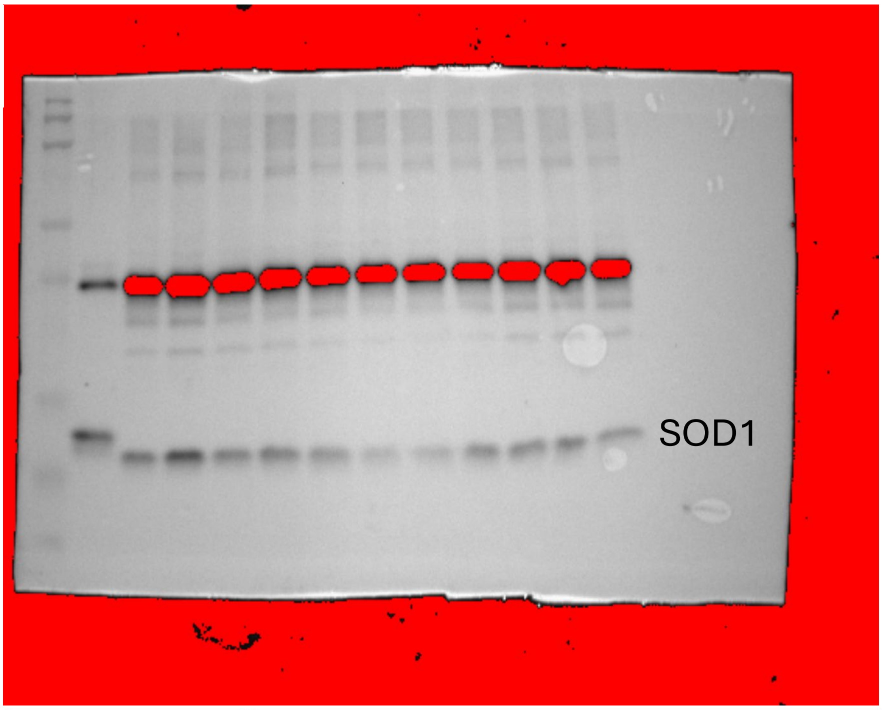

b

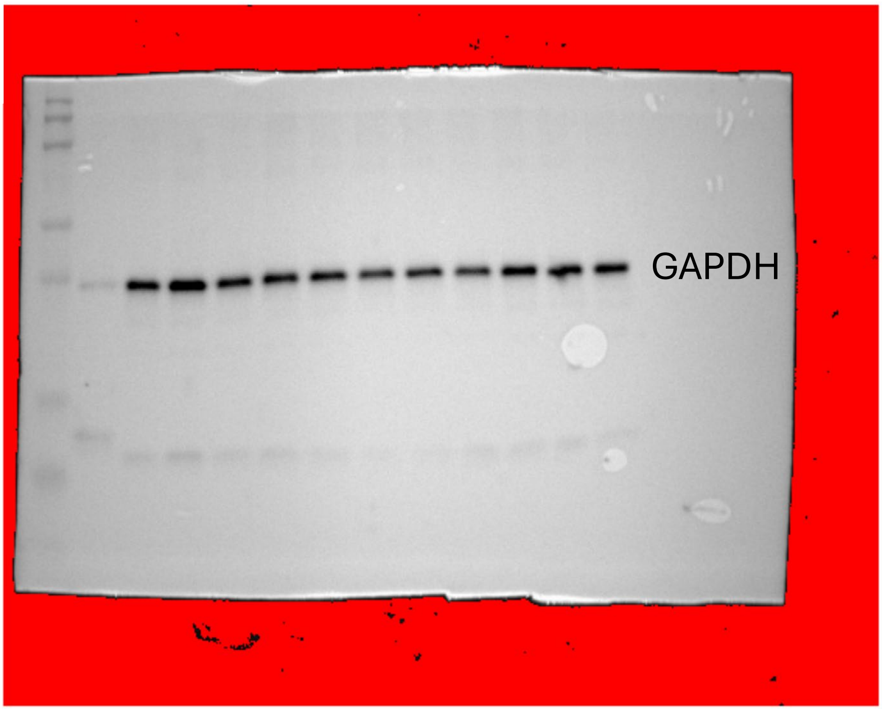

Supplementary Blot 1. Uncropped chemiluminescent blots of Gel 1. Blots were probed simultaneously for SOD1 (16kDa) and GAPDH (37kDa). Exposure and imaging was optimized for SOD1 (a) and GAPDH (b), with differences taken into account during image processing and densitometry.

a

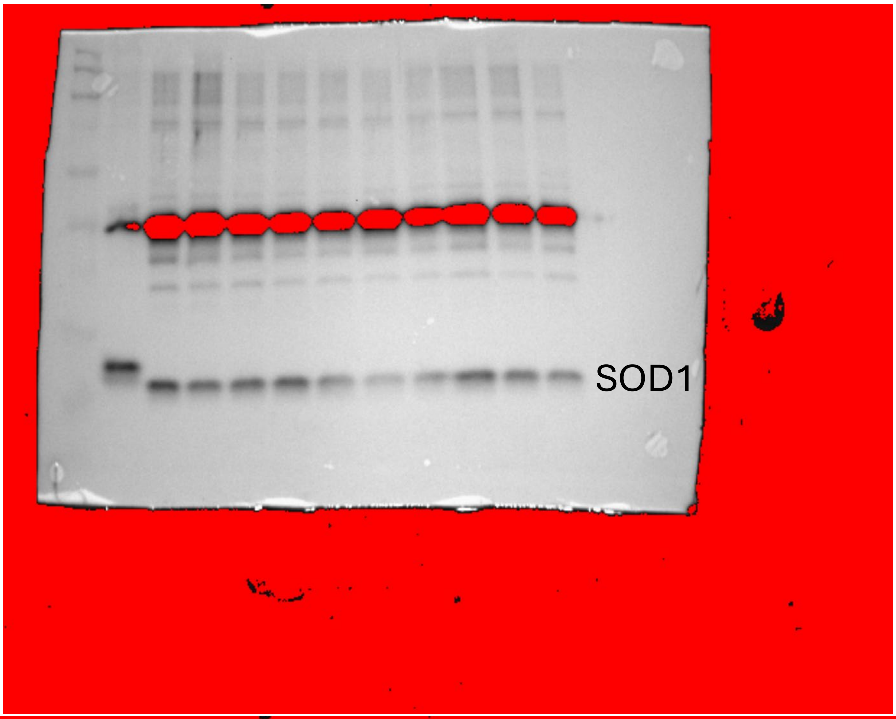

b

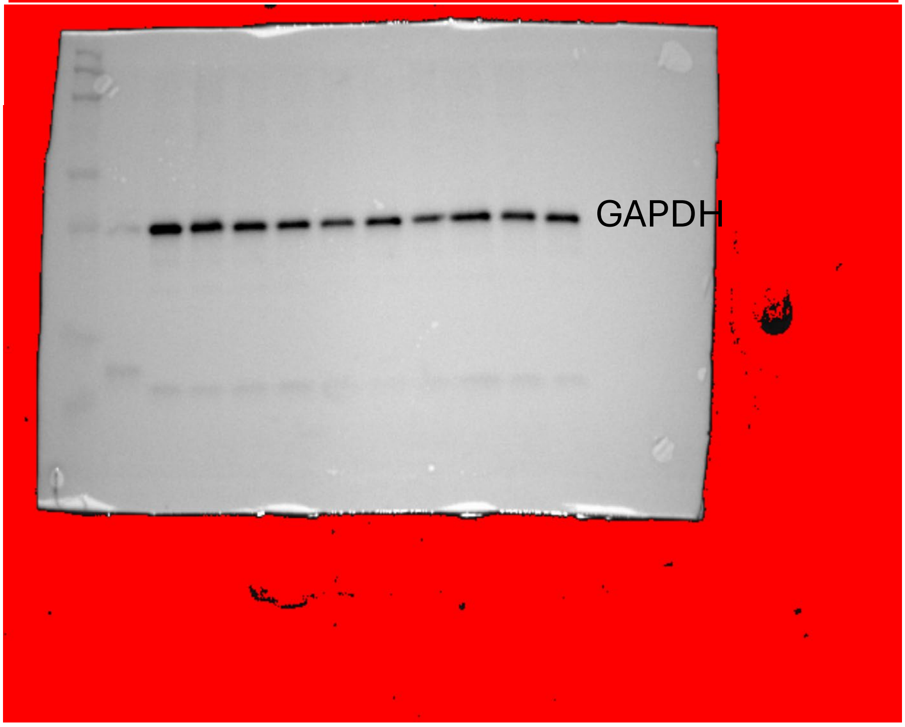

Supplementary Blot 2. Uncropped chemiluminescent blots of Gel 2. Blots were probed simultaneously for SOD1 (16kDa) and GAPDH (37kDa). Exposure and imaging was optimized for SOD1 (a) and GAPDH (b), with differences taken into account during image processing and densitometry.

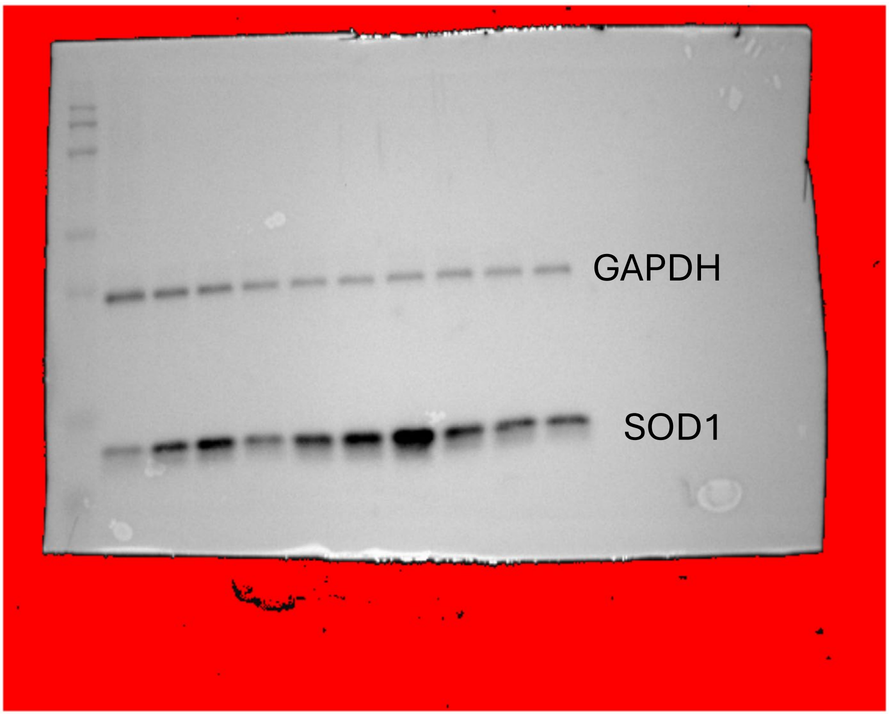

Supplementary Blot 3. Uncropped chemiluminescent blots of Gel 3. Blots were probed and imaged simultaneously for SOD1 (16kDa) and GAPDH (37kDa).

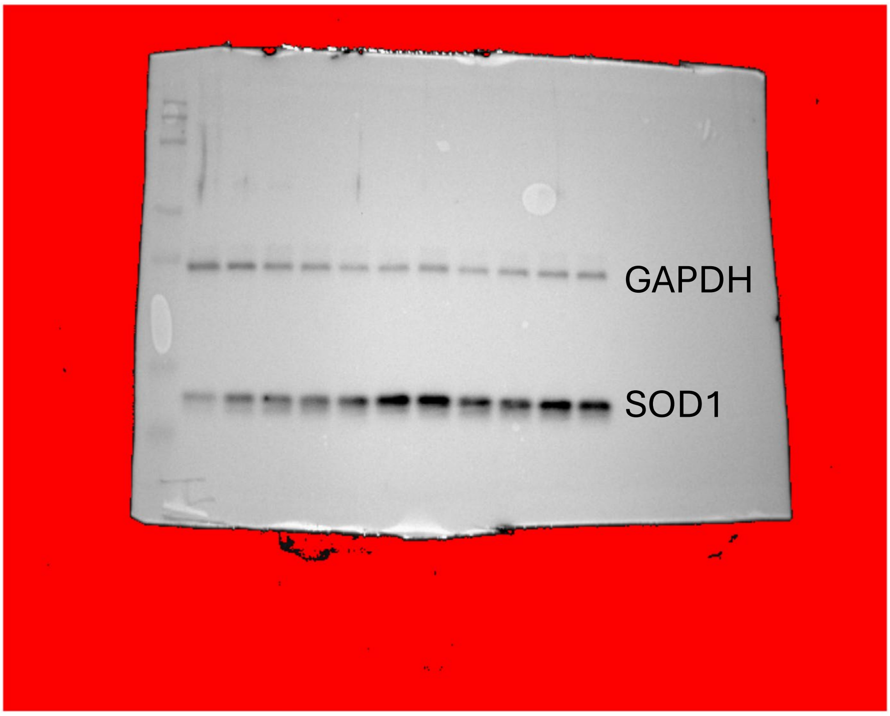

Supplementary Blot 4. Uncropped chemiluminescent blots of Gel 4. Blots were probed and imaged simultaneously for SOD1 (16kDa) and GAPDH (37kDa).

a

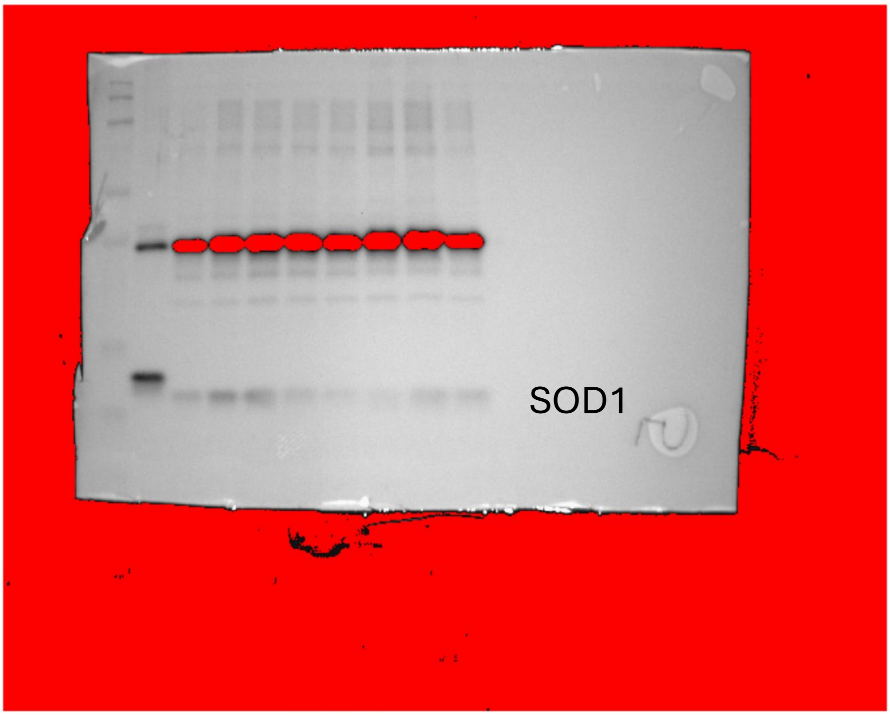

b

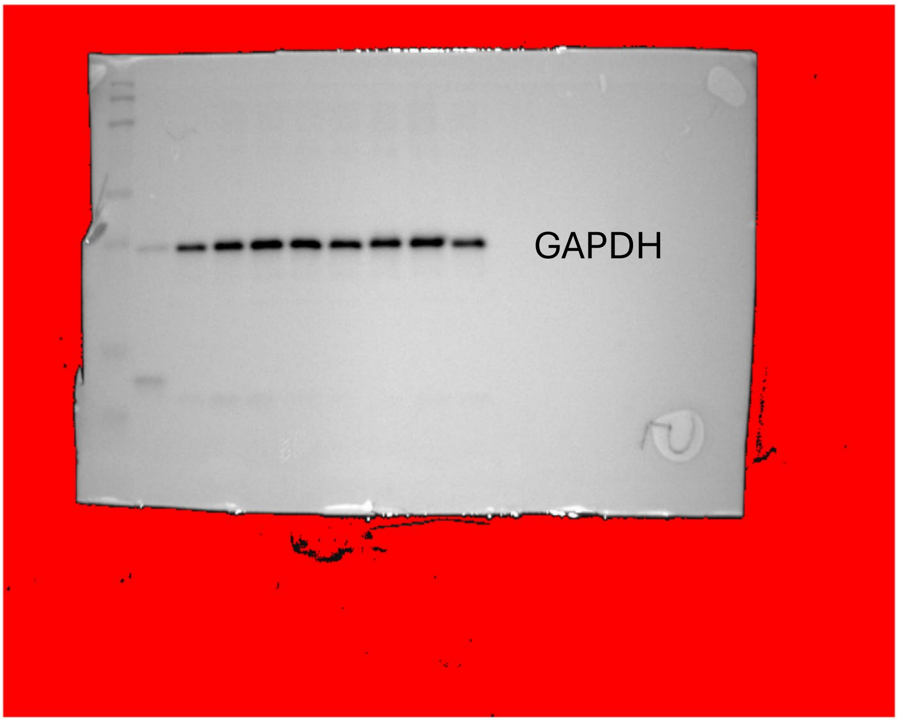

Supplementary Blot 5. Uncropped chemiluminescent blots of Gel 5. Blots were probed simultaneously for SOD1 (16kDa) and GAPDH (37kDa). Exposure and imaging was optimized for SOD1 (a) and GAPDH (b), with differences taken into account during image processing and densitometry.

a

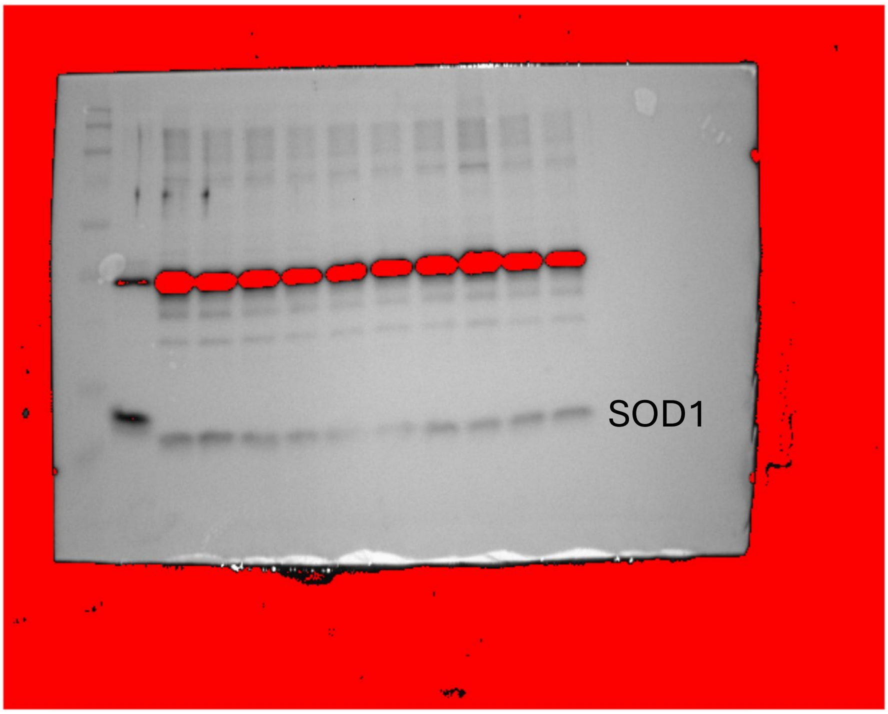

b

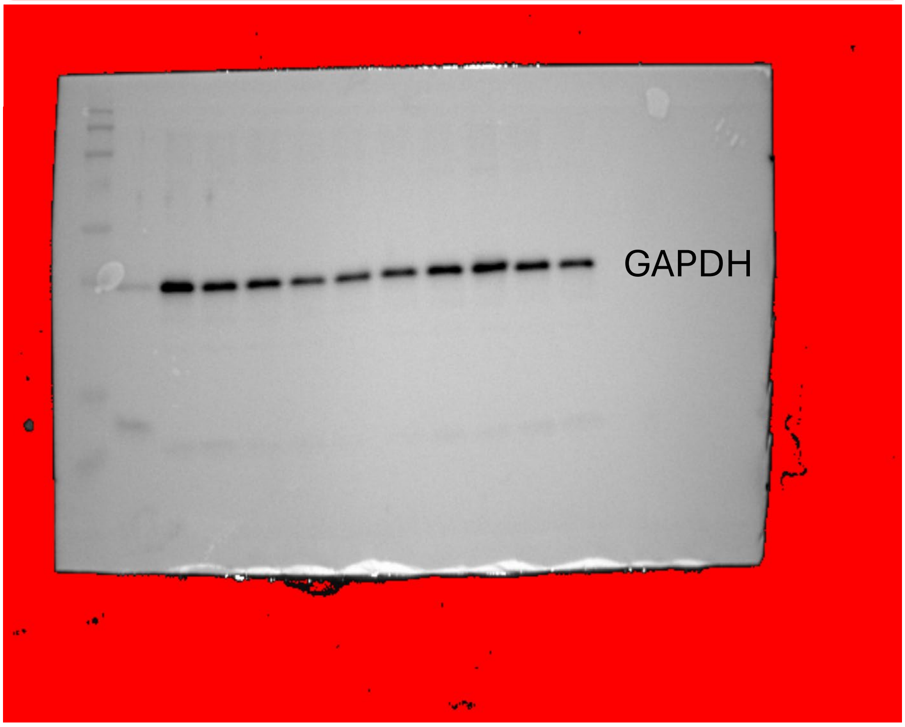

Supplementary Blot 6. Uncropped chemiluminescent blots of Gel 6. Blots were probed simultaneously for SOD1 (16kDa) and GAPDH (37kDa). Exposure and imaging was optimized for SOD1 (a) and GAPDH (b), with differences taken into account during image processing and densitometry.

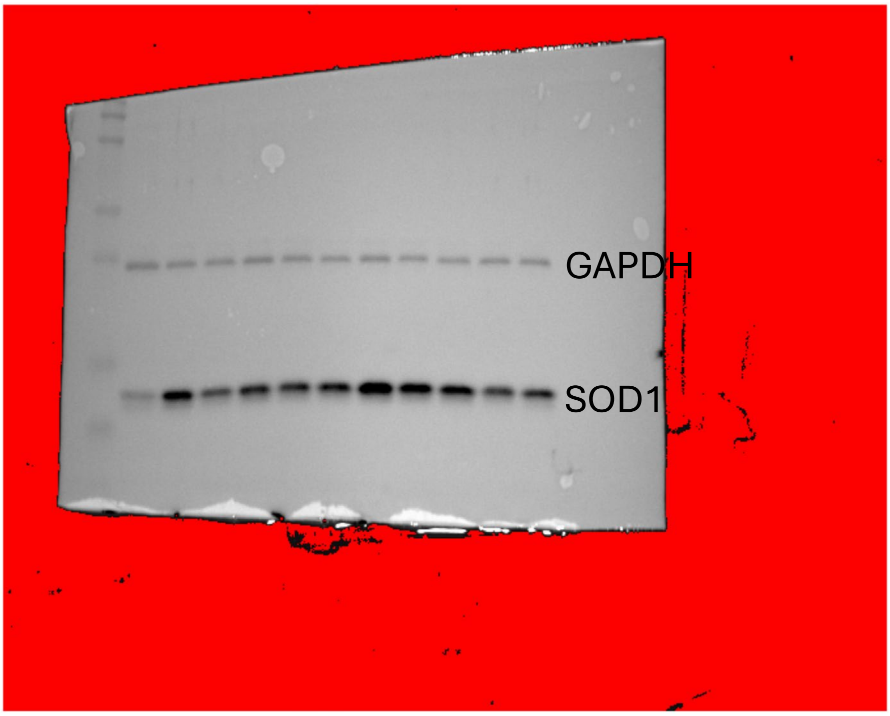

Supplementary Blot 7. Uncropped chemiluminescent blots of Gel 7. Blots were probed and imaged simultaneously for SOD1 (16kDa) and GAPDH (37kDa).

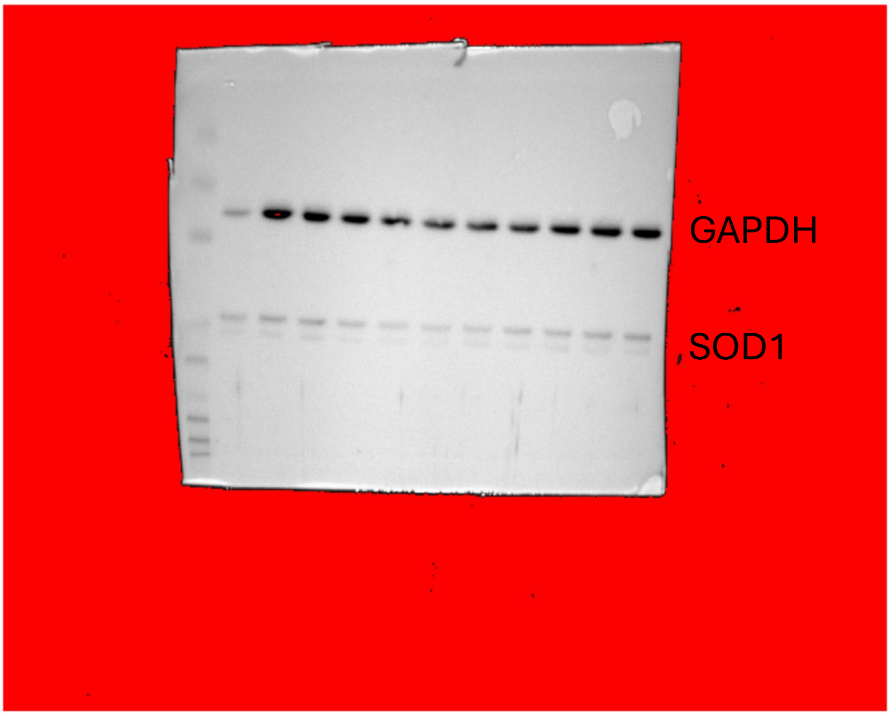

Supplementary Blot 8. Uncropped chemiluminescent blots of Gel 8. Blots were probed and imaged simultaneously for SOD1 (16kDa) and GAPDH (37kDa).
